# Public health round-up

**DOI:** 10.2471/BLT.24.011224

**Published:** 2024-12-01

**Climate and health**

A man walks away from a dry well in Lehele, Modogashe, Kenya, one of many places in East Africa hit hard by drought over the past three years. The drought has resulted in significant livestock and crop losses and high rates of malnutrition.

**Figure:**
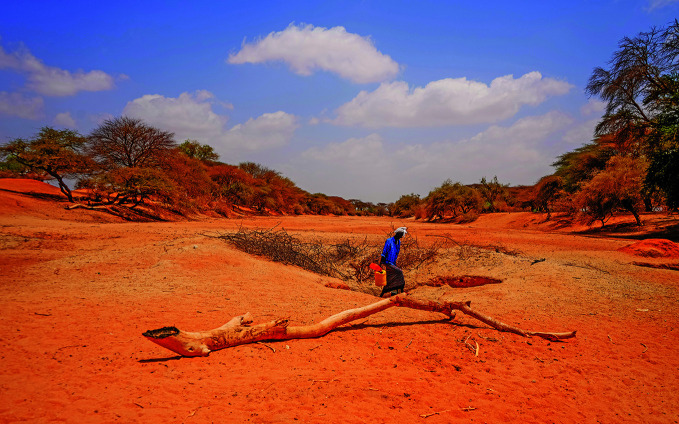
WHO/Billy Miaron.

## Gaza Strip emergency

The leaders of 15 United Nations and humanitarian organizations called for Israel to halt its assault on the Gaza Strip and to allow humanitarian aid to get through to the local population.

In a 1 November media release, the leaders and partners drew attention to worsening conditions in the Gaza Strip, highlighting the impact of daily bombardments by Israeli Defense Forces (IDF) – including bombardment of schools serving as shelters – the forced detainment of health workers and patients by the IDF, and the destruction of livestock and crops. According to the statement, the latter is threatening the region’s food systems, exposing the entire population to the risk of famine.

The leaders also called for the reversal of legislation recently enacted by Israel’s Knesset, designating the United Nations Relief and Works Agency for Palestine Refugees (UNRWA) a terrorist organization and prohibiting it from conducting any activities or providing services within Israel.

“If implemented, such measures would be a catastrophe for the humanitarian response in the Gaza Strip, diametrically opposed to the United Nations Charter, with potential dire impacts on the human rights of the millions of Palestinians depending on UNRWA’s assistance, and in violation of Israel’s obligations under international law,” the statement read.

Despite the appalling conditions in the Gaza Strip, the second round of the polio vaccination campaign in the Gaza Strip concluded on 5 November, reaching 556 774 children under 10 years with a second polio dose.

https://bit.ly/3O6KPDz

https://bit.ly/3O4BLiv

## Measles surges

Worldwide, there were an estimated 10.3 million cases of measles in 2023, a 20% increase from 2022. This is according to the latest global and regional estimates on measles cases and deaths published by the World Health Organization (WHO) and the U.S. Centers for Disease Control and Prevention (CDC) on 14 November. 

Inadequate immunization coverage is driving the increase, with more than 22 million children missing their first dose of measles vaccine in 2023. 

Globally, an estimated 83% of children received their first dose of measles vaccine last year, while only 74% received the recommended second dose.

Coverage of 95% or greater of two doses of measles vaccine is needed to prevent outbreaks.

https://bit.ly/4fvfaI8

## Diabetes rising

The number of adults living with diabetes worldwide has surpassed 800 million, more than quadrupling since 1990. This is according to a new study published in the *Lancet* on 14 November, World Diabetes Day. 

The study, highlights the scale of the diabetes epidemic and the urgent need for stronger global action to address both rising disease rates and widening treatment gaps, particularly in low- and middle-income countries (LMIC).

According to the study, global diabetes prevalence in adults rose from 7% to 14% between 1990 and 2022, with LMICs experiencing the largest increases. In 2022 an estimated 450 million adults aged 30 and older – about 59% of all adults with diabetes – went untreated.

https://bit.ly/4fqDFWW

## Tuberculosis burden up

Tuberculosis (TB) resurged as the highest-burden disease in the world. According to the *Global tuberculosis report 2024* published by WHO on 29 October, approximately 8.2 million people were newly diagnosed with the disease in 2023. This is the highest number recorded since WHO began global tuberculosis monitoring in 1995, and a significant increase from 7.5 million reported in 2022.

While the number of TB-related deaths decreased from 1.32 million in 2022 to 1.25 million in 2023, the total number of people falling ill with TB rose slightly to an estimated 10.8 million in 2023.

The report highlights mixed progress in the global fight against TB, along with persistent challenges that include significant underfunding.

https://bit.ly/4fHokRy

## Vaccine roll-out for mpox

The Access and Allocation Mechanism (AAM) for mpox allocated an initial 899 000 vaccine doses to 9 countries across the African region, with 85% of the doses going to the Democratic Republic of the Congo, which had reported 39 500 suspected cases as of mid-November, far more than any other country in the region.

The AAM is intended to ensure that the limited doses available are distributed effectively and fairly, with the overall objective being to control the different outbreaks.

The AAM principals from the Africa Centres for Disease Control and Prevention, the Coalition for Epidemic Preparedness Innovations; Gavi, the Vaccine Alliance; the United Nations Children’s Fund (UNICEF) and WHO approved the allocation, following the recommendations of an independent technical review committee of the continental incident management support team for mpox.

https://bit.ly/3O6egWr

## Vaccines for priority pathogens

WHO released a priority pathogen list for vaccine research and development. The list of 17 pathogens for which vaccines are urgently needed was published on 5 November as part of a WHO study – the first global effort to systematically prioritize endemic pathogens based on criteria that include regional disease burden, antimicrobial resistance risk and socioeconomic impact.

The list features familiar pathogens such as human immunodeficiency virus (HIV), but also includes pathogens that are increasingly resistant to antimicrobials such as Group A streptococci and *Klebsiella pneumoniae.*

The publication complements the WHO research and development blueprint for epidemics, which identifies priority pathogens that could cause future epidemics or pandemics.

https://bit.ly/3YPFwNZ

## Health emergency corps deployment

WHO and partners, in collaboration with Member States, activated the Global Health Emergency Corps (GHEC) for the first time to support countries facing mpox outbreaks.

The GHEC was activated in October, following the declaration of mpox as a public health emergency of international concern on 14 August.

As of 17 October, WHO had managed the deployment of 56 experts to the affected countries, including WHO staff, as well as experts mobilized through the Global Outbreak Alert and Response Network and the African Volunteers Health Corps.

https://bit.ly/3YInc9v

## Ending violence against children

More than 100 governments committed to ending violence against children, including nine pledging to ban corporal punishment – affecting an estimated 3 out of every 5 children regularly in their homes.

The commitments were made at the inaugural Ministerial Conference on Violence against Children which was held 7 – 8 November in Bogotá, Colombia.

Several countries committed to improving services for children experiencing violence or bullying, while others said they would invest in critical parenting support – one of the most effective interventions for reducing violence in the home.

https://bit.ly/4fqhHDi

**Cover photo**

A young woman delivers an educational talk about sexual and reproductive health to a group of adolescent girls in N'Djamena, Chad.

**Figure:**
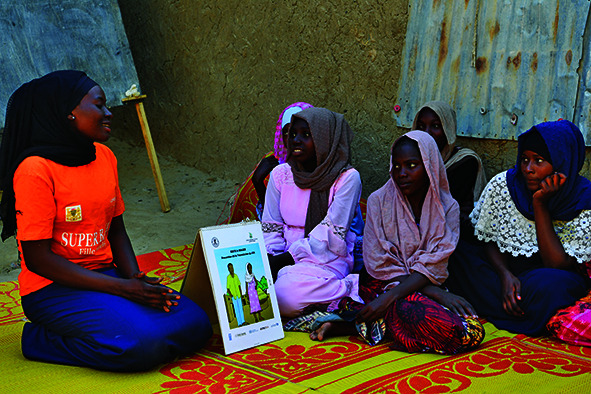
UNICEF/UN0583535/Dejongh

## Climate and health

WHO urged world leaders to adopt a more integrated approach to addressing climate change and health. The basis for the appeal was set out in the *COP29 special report on climate and health* which was released on 7 November, ahead of the 29th Conference of the Parties to the United Nations Framework Convention on Climate Change (COP29) which took place in Baku, Azerbaijan, from 11 to 22 November.

Developed by WHO in collaboration with partners, the report outlines critical policies needed to address issues related to people (especially the estimated 3.6 billion people who live in areas most susceptible to climate change impacts).

https://bit.ly/3CsctZm

## Egypt free of malaria

WHO certified Egypt as malaria-free, marking a major public health milestone for the nation of over 100 million people

In a 20 October statement Dr Hanan Balkhy, WHO Regional Director for the Eastern Mediterranean said, “This success in eliminating malaria is not just a victory for public health but a sign of hope for the entire world, especially for other endemic countries in our region.”

Egypt is the third country in the WHO Eastern Mediterranean Region, after the United Arab Emirates and Morocco, to achieve this certification, joining a global total of 44 countries and one territory.

https://bit.ly/3YNYcxl

**Looking ahead**

12 December. Universal Health Coverage & Universal Social Protection. Hybrid event. International Labour Organization, Geneva, Switzerland. https://bit.ly/3O7vBya

23 – 24 January 2025. International Conference on Global Health. London, United Kingdom. https://bit.ly/48PzwZW

28 January–2 February 2025. Prince Mahidol Award Conference 2025. Bangkok, Thailand. https://bit.ly/3AGb5BS

